# CoaSim: A flexible environment for simulating genetic data under coalescent models

**DOI:** 10.1186/1471-2105-6-252

**Published:** 2005-10-14

**Authors:** Thomas Mailund, Mikkel H Schierup, Christian NS Pedersen, Peter JM Mechlenborg, Jesper N Madsen, Leif Schauser

**Affiliations:** 1 Bioinformatics Research Center, University of Aarhus, Høegh Guldbergsgade 10, 8000 Århus C, Denmark; 2 Bioinformatics ApS, Høegh Guldbergsgade 10, 8000 Århus C, Denmark; 3 Department of Computer Science, University of Aarhus, Høegh Guldbergsgade 10, 8000 Århus C, Denmark

## Abstract

**Background:**

Coalescent simulations are playing a large role in interpreting large scale intra-specific sequence or polymorphism surveys and for planning and evaluating association studies. Coalescent simulations of data sets under different models can be compared to the actual data to test the importance of different evolutionary factors and thus get insight into these.

**Results:**

We have created the CoaSim application as a flexible environment for Monte Carlo simulation of various types of genetic data under equilibrium and non-equilibrium coalescent processes for a variety of applications. Interaction with the tool is through the Guile version of the Scheme scripting language. Scheme scripts for many standard and advanced applications are provided and these can easily be modified by the user for a much wider range of applications. A graphical user interface with less functionality and flexibility is also included. It is primarily intended as an exploratory and educational tool

**Conclusion:**

CoaSim is a powerful tool because of its flexibility and ease of use. This is illustrated through very varied uses of the application, e.g. evaluation of association mapping methods, parametric bootstrapping, and design and choice of markers for specific questions

## Background

These years witness popularity of coalescent based inference of evolutionary parameters, either through exact or approximate methods in order to understand within-species, and in particular human, evolution. This is fuelled by large scale efforts to collect data from different human populations, e.g. the HapMap [1] and ENCODE projects in humans. Important aims are to link genetic variation to phenotypic traits, e.g. complex disease, in order to identify the causal variants, as well as the human genetic history and dispersal.

The coalescent models are good null models to test specific hypotheses against, and therefore it is often useful to simulate genetic data under different coalescent models.

Coalescent simulation of data sets and subsequent calculation of some summary statistics on the data set can be used to obtain the distribution of this summary statistic under a given scenario, and this can be compared to the value of the summary statistic calculated from real data. Parametric bootstrapping can be done by estimating parameters from data under a given (coalescent) model, apply coalescent simulations under some model using the observed value of the parameters and see if the value observed is compatible with the simulation model. The efficiency of new methods can also be evaluated: Methods for inference of recombination rate and its variation, the importance of gene conversion versus recombination, as well as methods for association mapping under different genetic models have to rely on some heuristic approximations of the full likelihood models which are generally too computationally complex. Validating these approximations and inferring the importance of misspecification of the model of analysis employed is usually done using some variant of coalescent simulations.

Various applications for the simulation of the coalescent process are already available (e.g. [2-6]). The present application aims at attaining maximum flexibility in model specification. Population size changes, bottlenecks and demographics (migration rate matrix) can be specified precisely, there is a choice of mutation models (including microsatellite mutation models), recombination and gene conversion can be specified precisely and are allowed to vary over the region. Furthermore, complex disease models including arbitrary interactions between genes, varying penetrance and environmental effects, can also be specified. Case-control data sets under these disease models and any ascertainment scheme can then be generated for subsequent evaluation of association mapping methods aiming at detecting disease causing variants.

## Implementation

### Installation

CoaSim can be obtained at , where instructions for the installation are also provided. CoaSim is written in C++ and is available as source code and in a binary version for Linux operating system as rpm-files.

**Figure 1:**
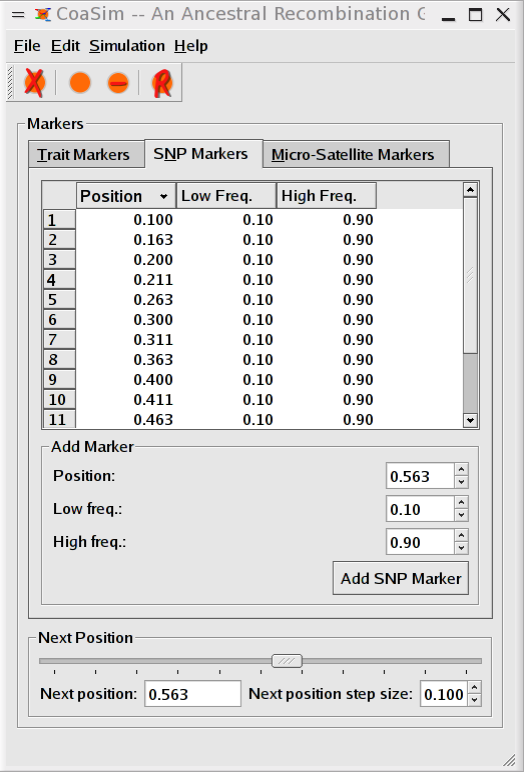
Screen shots from CoaSim GUI showing the input dialog.

**Figure 2:**
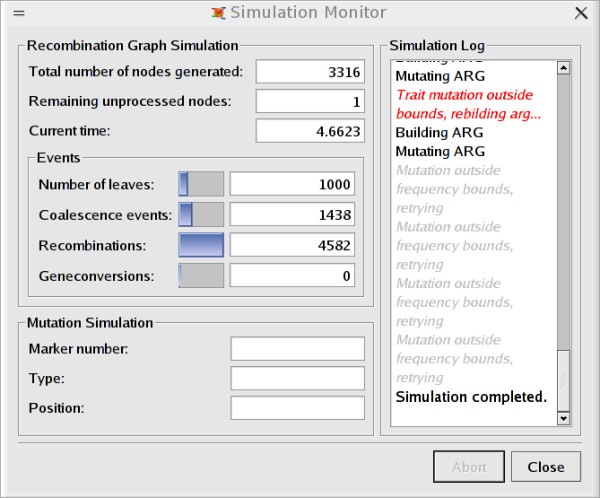
Screen shot from GUI showing the simulation status dialog.

**Figure 3:**
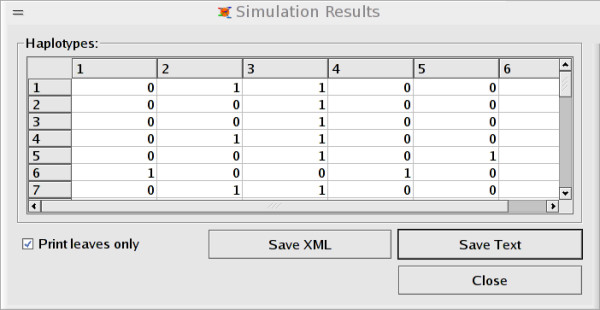
Screenshot from GUI showing simulated data.

An introduction to the simulator is provided as a "getting started" manual, where also the GUI version is described. The GUI is an intuitive interface to the simulator, allowing novice users to immediately start the simulations of sequences. Markers are added manually and the simulation stages can be monitored (see Figures 1, 2, 3). For more advanced purposes, users should turn to the guile-scheme version of CoaSim. Controlling the simulations through scheme makes CoaSim a very flexible and powerful simulation tool.

### Running the application

**Figure 4:**
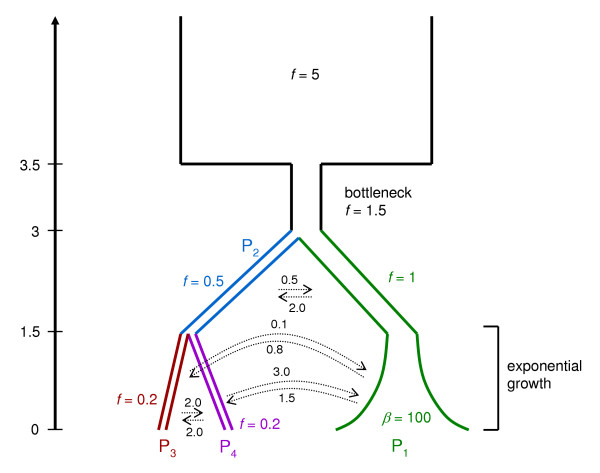
Example graphical representation of a demographic scenario with population splits, migration, and growth. *f *is the size of a population in units of 2*N*, β = 2*Nb *is the growth rate. Above the dotted arrows are the backwards migration rates, again scaled in units of 2*N*. Scheme code implementing the complete model is shown in Figure 5.

**Figure 5:**
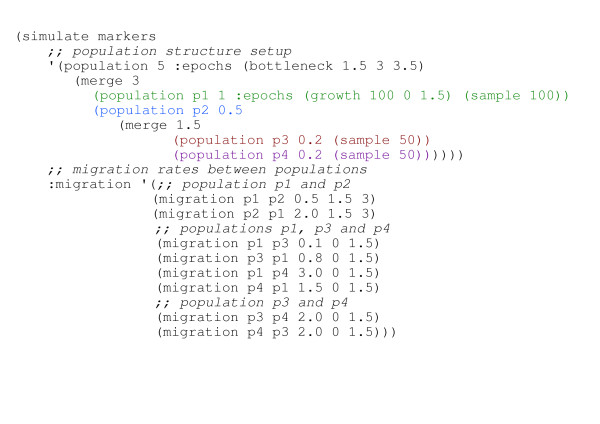
Example Scheme code that implements the demographic scenario of Figure 4. The Scheme code specifies both the population structures and the migration rates between populations, and simulates a sample of 100 individuals from population P_1 _and 50 from each of populations P_3 _and P_4_, with the merging of populations P_3 _and P_4 _into p_2 _at time 1.5 and the merging of populations P_1 _and P_2 _at time 3. The merge of P_1 _and P_2 _is followed by a bottleneck followed by a period of constant population size of f*2*N *= 10*N*.

The CoaSim Guile manual gives a number of examples of simple and advanced usage of the guile scheme version. Scripts that allow the iterative simulation of population samples under various demographic scenarios and disease models are introduced. An example demographic scenario is illustrated in Figure 4. Population merging times, sizes, migration rates etc. needs to be specified in the Scheme script. An example script that simulates the situation in Figure 4 is shown in Figure 5. The advanced usage of the tool for extracting summary statistics such as the mean of the total branch length of the simulated ARGs, the mean of the total tree height, the mean number of recombination nodes and coalescent nodes are all exemplified in scripts accompanying the distribution.

The output format of the sequence or marker data sets (such as Hudson's ms format) can be specified in the scheme script.

## Results and discussion

### Coalescent models

For an introduction to coalescent theory, see for example [7]. Basically, the ancestral history of a sample is simulated by repeated drawing of random numbers from competing exponential distributions corresponding to different evolutionary forces such as coalescence, migration, recombination, gene conversion. Coalescent intensities depend on the population size in each of demes where sampled genes are present. The number of demes, their sizes and the migration matrix are allowed to vary arbitrarily as specified by the input to the simulations. When an event (coalescence, migration, recombination or gene conversion) occurs, the state of the sample is updated and the intensities of the exponential distributions for the next event calculated. The process is continued until all parts of the gene have found a most recent common ancestor. The resulting graph, termed the ancestral recombination graph (ARG), is then used to generate data under different mutation models as specified by the user. Data can be generated either under the infinite sites or finite sites model of mutation. The marker positions can either be specified or chosen randomly, with uniform probability of distribution within the interval. It is possible either to condition on a mutation rate or on a given number of segregating sites for replicated simulations. The finite sites model can have any number *K *of states for a given position.

There are three kinds of built-in markers:

(1) Trait markers are binary polymorphisms (presence or absence of a trait, such as disease state) with a simple mutation model: after simulating the ARG, a mutation is placed uniformly at random on the tree local to the marker position, nodes below the mutation will have the mutant allele while all others will have the wild-type allele. A range of accepted mutant-frequencies can be specified and a simple rejection-sampling scheme is used to ensure it: if, after placing the mutation, the number of mutant leaves is not within the range, the ARG is rejected and the simulation restarted. This insures an unbiased collection of ARGs with a binary marker within a given frequency range.

(2) SNP markers resemble trait markers in that they are binary polymorphisms, and use the same mutation model as the trait-markers. They differ from the trait-markers in how the mutant-frequency is ensured: If, after the mutation has been placed, the number of mutant leaves does not fall within the accepted range, the mutation is re-placed, but the ARG is not rejected and re-simulated. This places a bias on the markers, but one that resembles the ascertainment bias seen in association studies, where SNPs are chosen to have frequencies in certain ranges. An unbiased sampling of mutations within the required frequency range can be obtained by constructing a new ARG and repeating the simulation process, until the required frequencies are attained.

(3) Microsatellite markers have a specified number of alleles, *k*, and a different mutation model than traits and SNPs. For microsatellite markers, each edge in the local tree at the marker is considered in turn and, with the likelihood of mutation being an exponential distribution that depends on the specified mutation rate and the length of the edge. If mutation occurs, a randomly chosen allele from 0 to *k*-1 is placed on the child node; if no mutation occurs, the child node gets a copy of the allele at the parent node.

In addition to the built-in marker types, Scheme scripting allows specification of most conceivable mutation models, the stepwise mutation model for microsatellites is included as an example.

Trait markers can be marked as being involved in disease susceptibility under a specified, but arbitrary model of disease risk as a function of biallelic variants at a number of sites specified a priori. A multidimensional matrix specifies disease risk for any combination of variants. Thus, from the simulated data and the disease model, case-control data of any size can be generated. Diploid data with known or unknown phase can be easily generated by calling the relevant function from the Scheme script as specified in the manual.

Splitting the sequences into cases and controls based solely on the allele at a trait marker or combination of alleles in a more complex disease determination is not always appropriate since it assumes full penetrance. However, in many complex diseases, penetrance is incomplete and dependent on environmental factors and general genetic background, and the same disease phenotypically can be caused by a different genetic pathway. Hence, it is possible to control the likelihood of a given individual belonging to the case or control group by specifying the probabilities given the possible genotypes.

Population size changes, bottlenecks, merging of populations (splitting of populations when viewed forward in time) and migration between sub-populations can vary arbitrarily as specified through the definitions of epochs in the Scheme script controlling the simulation (see Figures 4 and 5).

### Speed issues

We have measured execution times for a series of simulations. Simulating 10,000 SNP markers with a minor allele frequency of 10% for 10,000 chromosomes with a recombination rate of 100 took 85 seconds on a 3.0 GHz Pentium 4, 1 GB RAM machine. Increasing the recombination rate to 1000 caused the execution time to increase to 153 seconds.

We also simulated a scenario that in likely to find applications in simulating interacting disease loci. In this scenario, 1000 sequences were simulated for a setup where two trait markers which frequencies range between 20% to 40% were located in a region of 1000 SNP markers with a minor allele frequency of 10%,. This simulation took 28 seconds.

### Improvements to existing packages

Hudsons program ms provides many of CoaSims features for population growth, migration, recombination, gene conversion, arbitrary ascertainment schemes, recombination rate variation across the chromosome, stepwise mutation models, trait markers and cases and controls specified by the interaction between genes and genotype-specific penetrance probabilities. A difference is the ease of extracting the desired output. Using ms, the user has to post-process the output using custom made scripts, whereas CoaSim provides the required functionalities. With ms, it is not possible to specify the position of markers, to employ a finite sites mutation model or to simulate linked microsatellites.

### Some example applications of CoaSim

We have used CoaSim for three applications that demonstrate some of its flexibility.

1. Estimation of recombination rate and effective population size in Iceland from microsatellite data. In this study, microsatellite diploid, phase unknown, markers were simulated under different mutation models and for different rates of recombination. The expected decay of different measures of linkage disequilibrium with recombination rate could then be estimated and compared to the expected decay in a large microsatellite data set from Iceland. This allowed us to estimate the effective population size of the Icelandic population and to investigate whether the population bears any sign of recent population growth [8].

2. Case-control data sets were generated under a simple disease model (single locus dominance) but with various rates of heterogeneity and penetrance. The data with disease causing mutation removed was then directly piped into the GeneRecon program [9, 10] that attempts to infer the disease marker position from all the simulated markers. This allowed us to investigate the effect of marker density, penetrance, heterogeneity, number of cases/controls etc ([9], T. Mailund et al., unpublished results).

3. The effect of single recombination event. Genealogies were simulated for a given recombination rate but only genealogies with exactly one recombination event were used for simulation of marker (SNP) data sets. These data sets were then used as input to programs aimed at estimating the recombination rate, allowing us to estimate the variance in effect on data that a single recombination event can have and relate this to number of markers, population growth etc (M. H. Schierup et al. unpublished results).

## Conclusion

The CoaSim software package was designed for flexibility and with adaptations and extensions of the various Scheme scripts provided and the user manual, a wide range of situations can be accommodated. It provides the user with a much wider range of demographical models, of marker types and disease models without much loss of user friendliness compared to competing software.

## Availability and requirements

Project name: CoaSim 4.0

Project home page: 

Source codes for Guile and GUI versions are supplied [see Additional files]

Operating system(s): Linux Fedora 1–4, Redhat 9, and MacOSX

Programming language: C++ and scheme

Other requirements: Guile Scheme version 1.6. The GUI requires QT version 3.3

License: GNU

## Authors' contributions

TM, MHS, CNSP, JNM, LS, planned the project. TM, JNM and PJMM wrote the software, TM wrote the documentation. LS and TM tested the software. MHS, TM, LS and CNSP wrote the paper. All authors read and approved the final manuscript.

## Supplementary Material

Additional File 1 Click here for file

Additional File 2 Click here for file

Additional File 3 Click here for file

Additional File 4 Click here for file
