# Supplementary figures and images for: CoaSim: A flexible environment for simulating genetic data under coalescent models
Source: BMC Bioinformatics. 2005 Oct 14;6:252. doi: 10.1186/1471-2105-6-252 (PMC1274299; doi:10.1186/1471-2105-6-252)

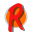

Supplement: Additional File 3 [file 1471-2105-6-252-S3.gz › coasim_gui/images/bioinformatics-run-icon.png]

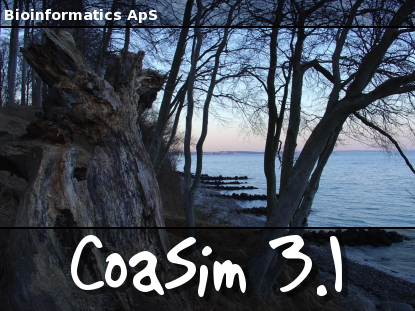

Supplement: Additional File 3 [file 1471-2105-6-252-S3.gz › coasim_gui/images/CoaSim-splash.png]

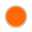

Supplement: Additional File 3 [file 1471-2105-6-252-S3.gz › coasim_gui/images/bioinformatics-clear-icon.png]

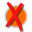

Supplement: Additional File 3 [file 1471-2105-6-252-S3.gz › coasim_gui/images/bioinformatics-exit-icon.png]

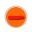

Supplement: Additional File 3 [file 1471-2105-6-252-S3.gz › coasim_gui/images/bioinformatics-delete-icon.png]

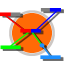

Supplement: Additional File 3 [file 1471-2105-6-252-S3.gz › coasim_gui/images/coasim-icon.png]
