# Supplementary material for: CoaSim: A flexible environment for simulating genetic data under coalescent models
Source: BMC Bioinformatics. 2005 Oct 14;6:252. doi: 10.1186/1471-2105-6-252 (PMC1274299; doi:10.1186/1471-2105-6-252)
Supplement: Additional File 4 [file 1471-2105-6-252-S4.gz › coasim-guile-4.0.0/doc/bindings.html]

xml version="1.0"?

CoaSim Scheme Bindings

# CoaSim specific scheme functions

## Main (coasim) module

- ancestral?
- bottleneck
- children
- coalescent-node?
- custom-marker
- event-time
- fold-nodes
- gene-conversion-end
- gene-conversion-node?
- gene-conversion-start
- growth
- interval->tree
- interval-end
- interval-start
- intervals
- leaf-node?
- local-trees
- migration
- ms-marker
- ms-marker?
- population-merge
- position
- recombination-node?
- recombination-point
- root
- sequences
- set-position!
- simulate
- simulate-sequences
- snp-marker
- snp-marker?
- total-branch-length
- trait-marker
- trait-marker?
- trapped?
- tree->interval
- tree-height

## Extra modules

**(coasim SNP genotypes)**

*This module contains functions for manipulating SNP (unphased) genotypes.*

- haplotypes->genotypes
- split-in-cases-controls-on-marker

**(coasim SNP haplotypes)**

*This module contains functions for manipulating SNP haplotypes.*

- split-in-cases-controls-on-marker

**(coasim batch)**

*This module contains functions for running batchs of simulations.
Although most of the functionallity in this module can be easily
implemented using Scheme's control-flow structures, the functions here
make batch running just a tad simpler.*

- fold
- repeat
- repeat-while
- repeat-with-index
- tabulate

**(coasim disease-modelling)**

*This module contains functions for disease models, specifically for
splitting simulated sequences in diseased and healthy individuals based
on their haplotypes or genotypes.*

- project-function
- qtl-on-markers
- remove-allele
- remove-alleles
- split-in-cases-controls
- split-in-cases-controls-on-marker
- split-in-cases-controls-on-markers
- split-on-probability
- split-on-threshold
- table->function

**(coasim io)**

*This module contains functions for reading and writing coasim data.*

- marker-positions-port
- marker-positions-printer
- positions-port
- positions-printer
- print-marker-positions
- print-positions
- print-sequences
- sequences-port
- sequences-printer

**(coasim markers)**

*This module contains functions manipulating sorted lists of markers.*

- insert-sorted
- insert-sorted-idx
- merge-markers
- rescale-markers!
- rescale-positions
- sort-markers

**(coasim rand)**

*This module contains functions making random marker configurations.*

- make-random-ms-markers
- make-random-positions
- make-random-snp-markers
- make-random-trait-markers

**(coasim statistics)**

*This module contains functions for calculating various statistics
on the ARG and on lists of haplotypes.*

- D
- S
- no-coalescence-events
- no-gene-conversions
- no-recombinations
- pi
- pi\_ij
- theta\_W

# Main (coasim) Module

## ancestral?

*Checks if a node contains ancestral material in a given point.*

#### Prototype

```
(ancestral? node point)
```

#### Example

```
(define coa-times '()) ; times for coalescent events at point 0.5
(define (coa-cb n k) 
  (if (ancestral? n 0.5) (set! coa-times (cons (event-time n) coa-times))))
(simulate markers no-leaves :rho 400 :coalescence-callback coa-cb)
```

### Details

Checks if a node contains ancestral material in a given point.

## bottleneck

*Makes a bottleneck epoch.*

#### Prototype

```
(bottleneck population scale-fraction begin-time end-time)
```

#### Example

```
(bottleneck 0 0.1 0.9 1.22)
```

### Details

Specify a bottleneck the simulation process will pass through.

The end-time parameter is optional, if left out the bottleneck
continues to infinity (or as long as the population exists).

## children

*Returns the children of a node.*

#### Prototype

```
(children node)
```

#### Example

```
(define (collect-event-times i)
  (letrec ((collect-et-r
	    (lambda (root point)
	      (if (not (ancestral? root point))
		  '()
		  (cond ((leaf-node? root) 
			 (list (event-time root)))
			((coalescent-node? root)
			 (let* ((cs (children root))
				(left (collect-et-r (car cs) point))
				(right (collect-et-r (cadr cs) point)))
			   (cons (event-time root) (append left right))))
			((or (recombination-node? root) 
			     (gene-conversion-node? root))
			 (cons (event-time root)
			       (collect-et-r (car (children root))
						point))))))))
    (collect-et-r (root i) (interval-start i))))
```

### Details

Returns the children of a node.

## coalescent-node?

*A predicate recognising coalescent nodes.*

#### Prototype

```
(coalescent-node? node)
```

#### Example

```
(if (coalescent-node? n) (event-time n))
```

### Details

Returns #t if `node` is a coalescent node, #f otherwise.

## custom-marker

*Creates a custom marker type*

#### Prototype

```
(custom-marker position ancestral-value mutation-function)
```

#### Example

```
(define (step-ms-marker pos theta)
  (let* ((msec (cdr (gettimeofday)))
         (random-state (seed->random-state msec))

         (waiting-time
          (lambda ()
            (let ((mean (/ 2 theta)));mean is 1/i where the intensity i is theta/2
              (* mean (random:exp random-state)))))

         (mutate-to
          (lambda (parent-allele)
            (if (< (random 1.0 random-state) 0.5)
                (- parent-allele 1)
                (+ parent-allele 1))))

         (mutate
          (lambda (parent child parent-allele)
            (let loop ((allele parent-allele)
                       (time-left 
                        (- (- (event-time parent) (event-time child))
                           (waiting-time))))
              (if (< time-left 0)
                  allele
                  (let ((new-allele (mutate-to allele))
                        (next-time (waiting-time)))
                    (loop new-allele (- time-left next-time))))))))

    (custom-marker pos 0 mutate)))
```

### Details

Creates a custom marker at position `position', with an
ancestral value given by `ancestral-value' (either an integer or
a thunk returning an integer), and a mutation function `mutate',
that takes arguments `parent-node' and `child-node' of an edge
to mutate, and the allele at the parent of the edge.

## event-time

*Returns the time of the event represented by a node.*

#### Prototype

```
(event-time node)
```

#### Example

```
(define coa-times '())
(define (coa-cb n k) (set! coa-times (cons (event-time n) coa-times)))
(define rc-times '())
(define (rc-cb n1 n2) (set! rc-times (cons (event-time n1) rc-times)))
(simulate markers no-leaves :rho 400
	  :coalescence-callback   coa-cb 
	  :recombination-callback rc-cb)
```

### Details

Returns the time of the event represented by a node.

## fold-nodes

*Calculate a value from all ARG nodes.*

#### Prototype

```
(fold-nodes arg f init)
```

#### Example

```
(define no-nodes
  (fold-nodes arg (lambda (n count) (+ count 1)) 0))
(define no-leaves
  (fold-nodes arg (lambda (n count) (if (leaf-node? n) (+ count 1) count)) 0))
```

### Details

Calculates a value from all ARG nodes. Call function `f' on each node
in the ARG, accumulating a value that is the result of the fold. For
each node, `f' is called with the node as its first argument and the
value calculated so far as its second argument; the second argument is
initially `init'.

## gene-conversion-end

*Returns the end point of a gene conversion.*

#### Prototype

```
(gene-conversion-end gene-conversion-node)
```

#### Example

```
(define gc-end '())
(define (gc-cb n1 n2 k) (set! gc-end (cons (gene-conversion-end n1) gc-end)))
(simulate markers no-leaves :gamma 10 :Q 0.2 :geneconversion-callback gc-cb)
```

### Details

Returns the end point of a gene conversion.

## gene-conversion-node?

*A predicate recognising gene conversion nodes.*

#### Prototype

```
(gene-conversion-node? node)
```

#### Example

```
(if (gene-conversion-node? n) (event-time n))
```

### Details

Returns #t if `node` is a gene conversion node, #f otherwise.

## gene-conversion-start

*Returns the start point of a gene conversion.*

#### Prototype

```
(gene-conversion-start gene-conversion-node)
```

#### Example

```
(define gc-start '())
(define (gc-cb n1 n2 k) (set! gc-start (cons (gene-conversion-start n1) gc-start)))
(simulate markers no-leaves :gamma 10 :Q 0.2 :geneconversion-callback gc-cb)
```

### Details

Returns the start point of a gene conversion.

## growth

*Makes a growth epoch.*

#### Prototype

```
(growth population beta begin-time end-time)
```

#### Example

```
(growth 0 10 0.9 1.22)
```

### Details

Specify a period of exponetial growth.

The end-time parameter is optional, if left out the bottleneck
continues to infinity (or as long as the population exists).

## interval->tree

*Returns tree local to an interval.*

#### Prototype

```
(interval->tree interval)
```

#### Example

```
(define markers (make-random-snp-markers 10 0.1 0.9))
(define intervals (intervals (simulate markers 100 :rho 400)))
(define trees (map interval->tree intervals))
```

### Details

Returns the tree local to an interval.

## interval-end

*Returns the end position of an interval.*

#### Prototype

```
(interval-end interval)
```

#### Example

```
(define markers (make-random-snp-markers 10 0.1 0.9))
(define intervals (let ((arg (simulate markers 100 :rho 400))) (intervals arg)))
(map interval-end intervals)
```

### Details

Returns the end position of an interval.

## interval-start

*Returns the start position of an interval.*

#### Prototype

```
(interval-start interval)
```

#### Example

```
(define p (arg-parameters rho Q G beta))
(define markers (make-random-snp-markers 10 0.1 0.9))
(define intervals (let ((arg (simulate p markers 100))) (intervals arg)))
(map interval-start intervals)
```

### Details

Returns the start position of an interval.

## intervals

*Returns the intervals sharing genealogy in the ARG as a list.*

#### Prototype

```
(intervals arg)
```

#### Example

```
(define markers (make-random-snp-markers 10 0.1 0.9))
(define intervals (let ((arg (simulate markers 100 :rho 400))) (intervals arg)))
```

### Details

Returns the intervals sharing genealogy in the ARG as a list.
Only intervals containing markers are returned.

## leaf-node?

*A predicate recognising leaf nodes.*

#### Prototype

```
(leaf-node? node)
```

#### Example

```
(if (leaf-node? n) 0.0 (event-time n))
```

### Details

Returns #t if `node` is a leaf node, #f otherwise.

## local-trees

*Returns the local trees, i.e. the trees for intervals sharing genealogy in the ARG as a list.*

#### Prototype

```
(local-trees arg)
```

#### Example

```
(define p (arg-parameters rho Q G beta))
(define markers (make-random-snp-markers 10 0.1 0.9))
(define trees (let ((arg (simulate markers 100 :rho 400))) (local-trees arg)))
```

### Details

Returns the local trees, i.e. the trees for
intervals sharing genealogy in the ARG as a list.
Only trees containing markers are returned.

## migration

*Makes a migration epoch.*

#### Prototype

```
(migration source-population destination-population rate begin-time end-time)
```

#### Example

```
(migration 0 1 0.1 0.9 1.22)
```

### Details

Specify a period of migration from population `source-population'
to `destination-population' with scaled migration rate `rate'.

## ms-marker

*Creates a micro-satellite marker on the simulated region.*

#### Prototype

```
(ms-marker position theta K)
```

#### Example

```
(ms-marker 0.5 0.2 10)
```

### Details

Creates a micro-satellite marker on the simulated region. The
first parameter determines the position (between 0 and 1) along
the region, the second the mutation rate of the marker, and the
third the size of the alphabet for the marker (in the K allele
model).

## ms-marker?

*A predicate that evalutes to true for micro-satellite markers only.*

#### Prototype

```
(ms-marker? marker)
```

#### Example

```
(ms-marker? marker)
```

### Details

A predicate that evalutes to true for micro-satellite markers and false
for all other types.

## population-merge

*Merges two sub-populations.*

#### Prototype

```
(population-merge merge-time pop1 pop2 ...)
```

#### Example

```
(population-merge 0.9 0 1)
```

### Details

Sets up the merge time for two or more sub-populations. The effect
of the merge is that all individuals in the populations are moved to the
first population.

## position

*Returns the position of a marker.*

#### Prototype

```
(position marker)
```

#### Example

```
(position marker)
```

### Details

Returns the position of a marker.

## recombination-node?

*A predicate recognising recombination nodes.*

#### Prototype

```
(recombination-node? node)
```

#### Example

```
(if (recombination-node? n) (event-time n))
```

### Details

Returns #t if `node` is a recombination node, #f otherwise.

## recombination-point

*Returns the recombination point of a recombination node.*

#### Prototype

```
(recombination-point recombination-node)
```

#### Example

```
(define rc-points '())
(define (rc-cb n1 n2 k) (set! rc-points (cons (recombination-point n1) rc-points)))
(simulate markers no-leaves :rho 400 :recombination-callback rc-cb)
```

### Details

Returns the recombination point of a recombination node.

## root

*Returns the root of the local tree of an interval.*

#### Prototype

```
(root interval-or-tree)
```

#### Example

```
 (use-modules (coasim rand))
 (define markers (make-random-snp-markers 10 0.1 0.9))
 (define trees (local-trees (simulate markers 100 :rho 400)))
 (map root trees)
```

### Details

Returns the root node of the local tree of an interval.
The argument to the function can be either a local interval, as returned
by the intervals function, or a local tree, as returned by the local-trees
function.

## sequences

*Returns the simulated sequences of an ARG as a list of lists.*

#### Prototype

```
(sequences arg)
```

#### Example

```
(define markers (make-random-snp-markers 10 0.1 0.9))
(define haplotypes (let ((arg (simulate markers 100 :rho 400))) (sequences arg)))
```

### Details

Returns the simulated sequences of an ARG as a list of lists.

## set-position!

*Changes the position of a marker.*

#### Prototype

```
(set-position! marker new-position)
```

#### Example

```
(set-position! marker 0.5)
```

### Details

Changes the position of a marker. In general, you will not need this
function, but it is useful when rescaling the interval to implement, e.g.
variable recombination rate.

## simulate

*Simulate an ARG and corresponding sequences.*

#### Prototype

```
(simulate marker-list sim-spec . additional-keyword-parameters)
```

#### Example

```
(define markers (make-random-snp-markers 10 0.1 0.9))
(define arg (simulate markers 100 :rho 400 :beta 10))

(define coa-times '())
(define (coa-cb n k) (set! coa-times (cons (event-time n) coa-times)))
(simulate markers 10 :coalescence-callback coa-cb :rho 400 :beta 10)
(display "coalescence times:\n")
(map (lambda (t) (display t)(newline)) coa-times)
(newline)
```

### Details

Simulate an ARG and corresponding sequences, based on ARG parameters,
a list of markers, and a simulation specification. The specification
is either a number--the number of samples to simulate -- or
a population structure specification -- see the *CoaSim Guile
Manual* for details.

The building of the ARG is affected by the following paramters, that
can be set using keyword arguments:

- **rho:** the *scaled recombination rate*, rho=4Nr. See
  e.g. *Hein, Schierup and Wiuf:* Gene Genealogies, Variation
  and Evolution, section 5.5 for details.
  By default, this parameter is 0.
- **gamma:** the *scaled gene-conversion rate*, gamma=4Ng.
  See e.g. *Hein, et al.:* section 5.10 for details.
  By default, this parameter is 0.
- **Q:** the *gene-conversion tract length intensity*.
  See e.g. *Hein, et al.:* section 5.10 for details.
  By default, this parameter is 0.
- **beta:** the *exponential growth rate*, beta=2Nb.
  See e.g. *Hein, et al.:* section 4.3 for details.
  By default, this parameter is 0.

For specifying migration rates between subpopulations (see the *CoaSim
Guile Manual* for details), the keyword parameter **:migrations**
is used. This parameter takes a list of migration specifications.

For fine-monitoring of the simulation, callback functions can be given
as key-word arguments. The supported callbacks are:

- **coalescence-callback:**
  called with the single node that is the result of a coalescent event,
  and the number of lineages at the time of the coalescent (i.e.
  the number of linages just after the event, moving forward in time).
- **recombination-callback:**
  called with the two nodes that is the result of a recombination
  event, and the number of lineages at the time of the recombination
  (i.e. the number of linages just after the event, moving forward
  in time).
- **geneconversion-callback:**
  called with the two nodes that is the result of a gene conversion
  event, and the number of lineages at the time of the gene conversion
  (i.e. the number of linages just after the event, moving forward
  in time).

The following additional keyword arguments are available:

- **keep-empty-intervals:** Turns off an optimisation that
  removes intervals not containing markers from the ARG simulation.
  This option is useful when the simulation is concerned with ARG
  properties rather than just the resulting sequences.
- **random-seed:** an integer used as random seed for the
  simulation. In most cases, this argument will not be used, but
  it is useful for regression testing of scheme modules for CoaSim.

## simulate-sequences

*Simulate a list of sequences.*

#### Prototype

```
(simulate-sequences markers no-leaves . additional-simulation-parameters)
```

#### Example

```
(define markers (make-random-snp-markers 10 0 1))
(define seqs (simulate-sequences markers 10 :rho 400))
```

### Details

Simulate a list of sequences. This function is just a short-cut
for `simulate' followed by `sequences':

```` ```
 (define (simulate-sequences . args)
   (sequences (apply simulate args)))
``` ````

## snp-marker

*Creates a SNP marker on the simulated region.*

#### Prototype

```
(snp-marker position low-freq high-freq)
```

#### Example

```
(snp-marker 0.5 0.1 0.9)
```

### Details

Creates a SNP marker on the simulated region. The first
parameter determines the position (between 0 and 1) along the
region, the second the lowest frequency accepted for the
mutation type, and the third the highest frequency accepted for the
mutation type.

## snp-marker?

*A predicate that evalutes to true for SNP markers only.*

#### Prototype

```
(snp-marker? marker)
```

#### Example

```
(snp-marker? marker)
```

### Details

A predicate that evalutes to true for SNP markers and false for
all other types.

## total-branch-length

*Returns the total tree branch length of the local tree of an interval.*

#### Prototype

```
(total-branch-length interval-or-tree)
```

#### Example

```
  (use-modules (coasim rand))
 (define markers (make-random-snp-markers 10 0.1 0.9))
 (define intervals (intervals (simulate markers 100 :rho 400)))
 (map total-branch-length intervals)
```

### Details

Returns the total tree branch length of the local tree of an interval.
The argument to the function can be either a local interval, as returned
by the intervals function, or a local tree, as returned by the local-trees
function.

## trait-marker

*Creates a trait marker on the simulated region.*

#### Prototype

```
(trait-marker position low-freq high-freq)
```

#### Example

```
(trait-marker 0.5 0.18 0.22)
```

### Details

Creates a trait marker on the simulated region. The first
parameter determines the position (between 0 and 1) along the
region, the second the lowest frequency accepted for the
mutation type, and the third the highest frequency accepted for the
mutation type.

## trait-marker?

*A predicate that evalutes to true for trait markers only.*

#### Prototype

```
(trait-marker? marker)
```

#### Example

```
(trait-marker? marker)
```

### Details

A predicate that evalutes to true for trait markers and false for
all other types.

## trapped?

*Checks if a node contains trapped material in a given point.*

#### Prototype

```
(trapped? node point)
```

#### Example

```
(define coa-times '()) ; times for coalescent events at point 0.5
(define (coa-cb n k) 
  (if (or (ancestral? n 0.5) (trapped? n 0.5))(set! coa-times (cons (event-time n) coa-times))))
(simulate markers no-leaves :rho 400 :coalescence-callback coa-cb)
```

### Details

Checks if a node contains trapped material in a given point.

## tree->interval

*Returns interval a local tree covers.*

#### Prototype

```
(tree->interval tree)
```

#### Example

```
(define markers (make-random-snp-markers 10 0.1 0.9))
(define intervals (let ((arg (simulate markers 100 :rho 400))) (intervals arg)))
(define trees (map interval->tree intervals)) 
(define intervals2 (map tree->interval trees))
```

### Details

Returns interval a local tree covers. The function is the reverse
of interval->tree.

## tree-height

*Returns the height of the local tree of an interval.*

#### Prototype

```
(tree-height interval-or-tree)
```

#### Example

```
 (use-modules (coasim rand))
 (define markers (make-random-snp-markers 10 0.1 0.9))
 (define intervals (local-trees (simulate markers 100 :rho 400)))
 (map tree-height intervals)
```

### Details

Returns the height of the local tree of an interval.
The argument to the function can be either a local interval, as returned
by the intervals function, or a local tree, as returned by the local-trees
function.

# (coasim SNP genotypes)

*This module contains functions for manipulating SNP (unphased) genotypes.*

## haplotypes->genotypes

*Translate a list of SNP haplotypes into a list of SNP genotypes.*

#### Prototype

```
 (haplotypes->genotypes haplotype-list)
```

#### Example

```
 (use-modules (coasim rand) (coasim SNP genotypes))
 (define haplotypes (simulate-sequences (make-random-snp-markers 4 0 1) 8))
 (display haplotypes)(newline)
 (define genotypes (haplotypes->genotypes haplotypes))
 (display genotypes)(newline)
```

### Details

Translate a list containing an even number of SNP haplotypes
into a list of SNP genotypes. The haplotypes are paired and the
alleles are translated such that homozygote 00 becomes 0, homozygote
11 becomes 1, and heterozygotes become 2.

## split-in-cases-controls-on-marker

*Split a list of genotypes into cases and controls.*

#### Prototype

```
(split-in-cases-controls-on-marker genotypes trait-idx)
```

#### Example

```
 (define haplotypes (simulate-sequences markers 100))
 (define genotypes (haplotypes->genotypes haplotypes))
 (define cases-and-controls (split-in-cases-controls-on-marker genotypes trait-idx
                                                     :disease-model 'recessive))
 (define cases    (car  cases-and-controls))
 (define controls (cadr cases-and-controls))
```

### Details

Split a dataset into cases and controls, based on the value at
trait-idx.

By default the alleles at the trait marker is removed from the resulting
lists; an optional parameter, :remove-trait, if set to #f, will prevent
removal of the trait marker.

The following keyword arguments can be used to control
the split:

- **homozygote-0-prob:** The probability that genotype 0 (or 00)
  is considered a case. By default this is 0.
- **homozygote-1-prob:** The probability that genotype 1 (or 11)
  is considered a case. By default this is 1.
- **heterozygote-prob:** The probability that genotype 2 (01 or 10)
  is considered a case. By default this is 0.5.
- **disase-model:** Should be either 'recessive or 'dominant;
  if 'dominant genotypes 1 (11) and 2 (01 or 10) are considered cases,
  if 'recessive only genotypes 1 (11) are considered cases.
  only
- **remove-trait:** If #t the alleles at the trait marker are
  removed, if #f they are kept.

# (coasim SNP haplotypes)

*This module contains functions for manipulating SNP haplotypes.*

## split-in-cases-controls-on-marker

*Split a list of haplotypes into cases and controls.*

#### Prototype

```
(split-in-cases-controls-on-marker haplotypes trait-idx)
```

#### Example

```
 (define haplotypes (simulate-sequences markers 100))
 (define cases-and-controls (split-in-cases-controls-on-marker haplotypes trait-idx))
 (define cases    (car  cases-and-controls))
 (define controls (cadr cases-and-controls))
```

### Details

Split a dataset into cases and controls, based on the value at
trait-idx.

By default the alleles at the trait marker is removed from the resulting
lists; an optional parameter, :remove-trait, if set to #f, will prevent
removal of the trait marker.

The following keyword arguments can be used to control
the split:

- **mutant-prob:** The probability that a mutant (allele 1) is
  considered a case. By default this is 1.
- **wild-type-prob:** The probability that a wild-type (allele 0)
  is considered a case. By default this is 0.
- **remove-trait:** If #t the alleles at the trait marker are
  removed, if #f they are kept.

# (coasim batch)

*This module contains functions for running batchs of simulations.
Although most of the functionallity in this module can be easily
implemented using Scheme's control-flow structures, the functions here
make batch running just a tad simpler.*

## fold

*Call a block of code a certain number of times, collecting the result
using a combination function.*

#### Prototype

```
(fold n comb init code)
```

#### Example

```
 (let* ((no-iterations 10000)
        (branch-sum
         (fold no-iterations (lambda (val sum) (+ val sum)) 0
          (let* ((m (snp-marker 0.5 0 1))
                 (arg (simulate (list m) 10))
                 (tree (car (local-trees arg)))
                 (branch-length (total-branch-length tree)))
            branch-length))))
   (display (/ branch-sum no-iterations))(newline))
```

### Details

Execute the code `code' `n' number of times,
combining the result of `code' using the `comb' function. The
`comb' is called with two arguments in each iteration, the first
is the result of having run `code', the second is the result of
the previous combination, initially the value `init' passed to
`fold'.

## repeat

*Call a block of code a certain number of times.*

#### Prototype

```
(repeat n c)
```

#### Example

```
 (use-modules (coasim batch))
 (repeat 20
  (let* ((m (snp-marker 0.5 0 1))
         (arg (simulate (list m) 10 :random-seed 10))
         (tree (car (local-trees arg))))
    (display tree)))
 (newline)
```

### Details

Execute the code `c' `n' number of times.

## repeat-while

*Call a block of code until a predicate evaluates to false.*

#### Prototype

```
(repeat-while p c)
```

#### Example

```
 (use-modules (ice-9 format) (coasim batch))

 (repeat-while (lambda (branch-length) (< branch-length 5))
  (let* ((m (snp-marker 0.5 0 1))
         (arg (simulate (list m) 10))
         (tree (car (local-trees arg)))
         (branch-length (total-branch-length tree)))
    (format #t "~4f " branch-length)
    branch-length))
 (newline)
```

### Details

Execute the code `c' until the predicate `p', evaluated on
the result of `c' evaluates to false.

## repeat-with-index

*Call a block of code a certain number of times.*

#### Prototype

```
 (repeat-with-index (i n) c)
 (repeat-with-index (i start stop) c)
 (repeat-with-index (i start stop step) c)
```

#### Example

```
 (use-modules (coasim batch))
 (repeat-with-index (i 10)
    (let* ((markers (make-random-snp-markers 10 0 1))
           (seqs (simulate-sequences markers 10))
           (pos-file (string-append "positions." (number->string i) ".txt"))
           (seq-file (string-append "sequences." (number->string i) ".txt")))
      (call-with-output-file pos-file (marker-positions-printer markers))
      (call-with-output-file seq-file (sequences-printer seqs))))
 
 (repeat-with-index (i 2 12)
    (let* ((markers (make-random-snp-markers 10 0 1))
           (seqs (simulate-sequences markers 10))
           (pos-file (string-append "positions." (number->string i) ".txt"))
           (seq-file (string-append "sequences." (number->string i) ".txt")))
      (call-with-output-file pos-file (marker-positions-printer markers))
      (call-with-output-file seq-file (sequences-printer seqs))))
 
 (repeat-with-index (i 2 20 2)
    (let* ((markers (make-random-snp-markers 10 0 1))
           (seqs (simulate-sequences markers 10))
           (pos-file (string-append "positions." (number->string i) ".txt"))
           (seq-file (string-append "sequences." (number->string i) ".txt")))
      (call-with-output-file pos-file (marker-positions-printer markers))
      (call-with-output-file seq-file (sequences-printer seqs))))
```

### Details

Execute the code `c' a number of times, making the index
variable `i' visible to the code in `c'. Comes in three
variants, a simple iteration from 1 to `n', an iteration from
`start' to `stop', and an iteration from `start' to `stop' in
jumps of `step'.

## tabulate

*Call a block of code a certain number of times, collecting
the results in a list.*

#### Prototype

```
 (tabulate n c)
```

#### Example

```
 (use-modules (coasim batch))
 (let* ((no-iterations 10000)
        (branch-lengths
         (tabulate no-iterations
          (let* ((m (snp-marker 0.5 0 1))
                 (arg (simulate (list m) 10))
                 (tree (car (local-trees arg)))
                 (branch-length (total-branch-length tree)))
            branch-length))))
   (display (/ (apply + branch-lengths) no-iterations))(newline))
```

### Details

Execute the code `c' `n' number of times, collecting the
results of evaluating `c' in a list that is returned as the
result of the tabulation.

# (coasim disease-modelling)

*This module contains functions for disease models, specifically for
splitting simulated sequences in diseased and healthy individuals based
on their haplotypes or genotypes.*

## project-function

*Projects a list of allels to a subset for a function call.*

#### Prototype

```
(project-function f indices)
```

#### Example

```
 (use-modules (coasim disease-modelling))
 (define is-case?
   (project-function (lambda (a1 a3) (= a1 1) (= a3 0)) '(1 3)))
```

### Details

Translates a function that takes a subset of allels as input, into a
function that takes the full list of alleles.

If function f takes, say, two alleles as input, and we want to call
it on index 1 and 3, we can use function (project-function f '(1 3))
that will work on the full allele list by calling f with allele 1
and 3.

## qtl-on-markers

*Calculates quantitative values for haplotypes based on selected
alleles.*

#### Prototype

```
 (qtl-on-markers sequences marker-indices f)
```

#### Example

```
 (use-modules (coasim disease-modelling))
 (let ((qtl-value (lambda (a2 a4) (+ (* 2 a2) a4)))))
     (qtl-on-markers seqs '(2 4) qtl-value)))
```

### Details

Calculate a quantitative value for each sequence in sequences
by calling f with the alleles at marker-indices for each sequence.

Returns a list of lists, where the car is the sequence and the
cadr is the calculated value.

By default the alleles at the marker indices are removed from the
resulting lists; an optional parameter, :remove-traits, if set to #f,
will prevent removal of the marker alleles.

## remove-allele

*Removes the alleles of the marker at `idx' from the sequences.*

#### Prototype

```
(remove-allele sequences idx)
```

#### Example

```
 (use-modules (coasim disease-modelling))
 (remove-allele seqs trait-idx)))
```

### Details

Removes the alleles at `idx' from the sequences; useful for example for
removing a trait marker from a dataset.

## remove-alleles

*Removes the alleles of the markers at `indices' from the sequences.*

#### Prototype

```
(remove-alleles sequences indices)
```

#### Example

```
 (use-modules (coasim disease-modelling))
 (remove-allele seqs trait-indices)))
```

### Details

Removes the alleles at `inidices' from the sequences;
useful for example for removing the trait markers from a dataset.

## split-in-cases-controls

*Split a list of sequences into cases and controls.*

#### Prototype

```
 (split-in-cases-controls sequences is-case?)
```

#### Example

```
 (use-modules (coasim disease-modelling))
 (let* ((is-mutant? (lambda (a) (= 1 a)))
        (allele     (lambda (h idx) (list-ref h idx)))
        (is-case?   (lambda (h) (and (is-mutant? (allele h 2))
                                     (is-mutant? (allele h 4))))))
     (split-in-cases-controls seqs is-case?))
```

### Details

Split a dataset into cases and controls, based on the
predicate is-case?. If a sequence satisfy the is-case? predicate,
the sequence is considered a case, otherwise a control.

## split-in-cases-controls-on-marker

*Split a list of sequences into cases and controls.*

#### Prototype

```
 (split-in-cases-controls-on-marker sequences marker-idx is-case?)
```

#### Example

```
 (use-modules (coasim disease-modelling))
 (let ((is-case? (lambda (a) (= 1 a))))
     (split-in-cases-controls-on-marker seqs marker-idx is-case?)))
```

### Details

Split a dataset into cases and controls, based on the value of the
marker at index marker-idx. If the value at that index satisfy the
is-case? predicate, the sequence is considered a case, otherwise a
control.

By default the alleles at the marker is removed from the resulting
lists; an optional parameter, :remove-trait, if set to #f, will prevent
removal of the marker alleles.

## split-in-cases-controls-on-markers

*Split a list of sequences into cases and controls.*

#### Prototype

```
 (split-in-cases-controls-on-markers sequences marker-indices is-case?)
```

#### Example

```
 (use-modules (coasim disease-modelling))
 (let ((is-case? (lambda (a2 a4) (and (= 1 a2) (= 1 a4)))))
     (split-in-cases-controls-on-markers seqs '(2 4) is-case?)))
```

### Details

Split a dataset into cases and controls, based on the values of the
alleles at indices marker-indices. If thes value at those indices
satisfy the is-case? predicate, the sequence is considered a case,
otherwise a control.

By default the alleles at the marker indices are removed from the
resulting lists; an optional parameter, :remove-traits, if set to #f,
will prevent removal of the marker alleles.

## split-on-probability

*Split a list of sequences with associated probabilities based on
the probability.*

#### Prototype

```
 (split-on-probability sequence-probability-list)
```

#### Example

```
 (use-modules (coasim disease-modelling))
 (let* ((qtl-value (lambda (a2 a4) (+ (* .2 a2) (* .1 a4))))
        (qtl-seqs (qtl-on-markers seqs '(2 4) qtl-value))
        (cases-controls (split-on-probability qtl-seqs))
        (cases (car cases-controls))
        (controls (cadr cases-controls)))
    ...)
```

### Details

Split a list of sequences with associated probabilities.

The input is a list of lists, where the first element in each list is
a sequence and the second is a probability. The sequences are
separated into two output lists; for pair '(seq p), seq
is put in the first list with probability p and in the second with
probability 1-q.

## split-on-threshold

*Split a list of sequences with associated quantitative values based on
a threshold for the value.*

#### Prototype

```
 (split-on-threshold sequence-qtl-list threshold)
```

#### Example

```
 (use-modules (coasim disease-modelling))
 (let* ((qtl-value (lambda (a2 a4) (+ (* 2 a2) a4)))
        (qtl-seqs (qtl-on-markers seqs '(2 4) qtl-value))
        (cases-controls (split-on-threshold qtl-seqs 1.5))
        (cases (car cases-controls))
        (controls (cadr cases-controls)))
    ...)
```

### Details

Split a list of sequences with associated quantitative values based on
a threshold for the value.

The input is a list of lists, where the first element in each list is
a sequence and the second is a quantitative value. This is the kind
of values that qtl-on-markers return.

The result is a list where the car contains the sequences where the
associated value was below the threshold and where the cadr contains
the sequences where the associated value was equal to or above the
threshold.

## table->function

*Translates a table into a function usable by e.g. the qtl-on-markers
function.*

#### Prototype

```
(table->function table)
```

#### Example

```
 (use-modules (coasim disease-modelling))
 (define f-hash
   (let ((h (make-hash-table 4)))
     (hash-create-handle! h '(0 0)  0)
     (hash-create-handle! h '(0 1)  0)
     (hash-create-handle! h '(1 0) .1)
     (hash-create-handle! h '(1 1) .5)
     (table->function h)))
 
 (define f-alist
   (let ((t
 	 (acons '(0 0)  0
 	 (acons '(0 1)  0
 	 (acons '(1 0) .1
 	 (acons '(1 1) .5
 	 '()))))))
     (table->function t)))
```

### Details

Translates a table, either a hash table or an associative list, into
a function that can be used with qtl-on-markers or
split-in-cases-controls-on-markers.

# (coasim io)

*This module contains functions for reading and writing coasim data.*

## marker-positions-port

*Convenience function for printing marker-positions.*

#### Prototype

```
(marker-positions-port port)
```

#### Example

```
(map (position-port port) list-of-marker-positions)
```

### Details

A convenience function, based on `print-marker-positions', that, given a
list a port, gives a function with one argument, a position, that
when applied writes the marker-positions to the port.

The function is useful for mapping over lists of marker-positions.

## marker-positions-printer

*Convenience function for printing marker-positions.*

#### Prototype

```
(marker-positions-printer marker-positions)
```

#### Example

```
(call-with-output-file "marker-positions.txt" (marker-positions-printer marker-positions))
```

### Details

A convenience function, based on `print-marker-positions', that, given a
list of marker-positions, gives a function with one argument, a port, that
when applied writes the marker-positions to the port.

The function is useful for calls to `call-with-output-file'.

## positions-port

*Convenience function for printing positions.*

#### Prototype

```
(positions-port port)
```

#### Example

```
(map (position-port port) list-of-positions)
```

### Details

A convenience function, based on `print-positions', that, given a
list a port, gives a function with one argument, a position, that
when applied writes the positions to the port.

The function is useful for mapping over lists of positions.

## positions-printer

*Convenience function for printing positions.*

#### Prototype

```
(positions-printer positions)
```

#### Example

```
(call-with-output-file "positions.txt" (positions-printer positions))
```

### Details

A convenience function, based on `print-positions', that, given a
list of positions, gives a function with one argument, a port, that
when applied writes the positions to the port.

The function is useful for calls to `call-with-output-file'.

## print-marker-positions

*Print a list of positions from markers.*

#### Prototype

```
(print-marker-positions port markers)
```

#### Example

```
(print-marker-positions (current-output-port) markers)
```

### Details

This function prints the positions of the markers in the list `markers'
to the output port `port'.

## print-positions

*Print a list of positions.*

#### Prototype

```
(print-positions port positions)
```

#### Example

```
(print-positions (current-output-port) positions)
```

### Details

This function prints the positions (numbers) in the list `positions' to
the output port `port'.

## print-sequences

*Print a list of sequences.*

#### Prototype

```
(print-sequences port sequences)
```

#### Example

```
(print-sequences (current-output-port) sequences)
```

### Details

This function prints the sequences in the list `sequences' to
the output port `port'.

## sequences-port

*Convenience function for printing sequences.*

#### Prototype

```
(sequences-port port)
```

#### Example

```
(map (sequence-port port) list-of-sequences)
```

### Details

A convenience function, based on `print-sequences', that, given a
list a port, gives a function with one argument, a sequence, that
when applied writes the sequences to the port.

The function is useful for mapping over lists of sequences.

## sequences-printer

*Convenience function for printing sequences.*

#### Prototype

```
(sequences-printer sequences)
```

#### Example

```
(call-with-output-file "sequences.txt" (sequences-printer sequences))
```

### Details

A convenience function, based on `print-sequences', that, given a
list of sequences, gives a function with one argument, a port, that
when applied writes the sequences to the port.

The function is useful for calls to `call-with-output-file'.

# (coasim markers)

*This module contains functions manipulating sorted lists of markers.*

## insert-sorted

*Inserts a marker in a sorted list of markers.*

#### Prototype

```
(insert-sorted sorted-marker-list marker)
```

#### Example

```
(insert-sorted sorted-marker-list marker)
```

### Details

This function inserts a marker into a sorted list of markers and
return the resulting list.

## insert-sorted-idx

*Inserts a marker in a sorted list of markers..*

#### Prototype

```
(insert-sorted-idx marker-list marker)
```

#### Example

```
 (define snp-markers (make-random-snp-markers 10 0.1 0.9))
 (define disease-marker (car (make-random-trait-markers 1 0.18 0.22)))
 (define markers-and-index (insert-sorted-idx snp-markers disease-marker))
```

### Details

This function inserts a marker into a sorted list of markers and
return a list who's first element is the resulting list and who's
second element is the index the marker got in the new list.

## merge-markers

*Merge two or more lists of sorted markers.*

#### Prototype

```
(merge-markers . list-of-marker-lists)
```

#### Example

```
(merge-markers marker-list-1 marker-list-2 marker-list-3)
```

### Details

Merge two or more lists of sorted markers.

## rescale-markers!

*Rescales marker positions to simulate variable recombination rate.*

#### Prototype

```
(rescale-markers! markers recombination-rates)
```

#### Example

```
 (use-modules (coasim markers))
 (define recomb-rates '((0.5 40) (0.25 400) (0.25 40)))
 (define scaled (rescale-markers! markers recomb-rates))
```

### Details

Rescales the positions of the markers in `markers' according to
`recombination-rates',
a list of pairs where the first element is a lenght (of the 0-1 interval)
and the second is the recombination rate over that range. (The actual
value of the rate is not important, only the relative differences
between the rates in the list).

Similar to the function `rescale-positions', except that for this
function the markers are moved according to the rescaling.

## rescale-positions

*Rescales marker positions to simulate variable recombination rate.*

#### Prototype

```
(rescale-positions positions recombination-rates)
```

#### Example

```
 (use-modules (coasim markers))
 (define positions '(0 0.1 0.2 0.3 0.4 0.5 0.6 0.7 0.8 0.9))
 (define recomb-rates '((0.5 40) (0.25 400) (0.25 40)))
 (define new-positions (rescale-positions positions recomb-rates))
```

### Details

Rescales the positions in `positions' according to `recombination-rates',
a list of pairs where the first element is a lenght (of the 0-1 interval)
and the second is the recombination rate over that range. (The actual
value of the rate is not important, only the relative differences
between the rates in the list).

The `recombination-rates' list is translated into a piece-wise linear
function, where the different regions of the 0-1 interval specified by
the distances in the list have different slope

Let *li* be length *i* in
`recombination-rages', and let *bpi* be the sum of
the first *i* lengths:
*Σj<i li*.
Let *ai* be rate
*i* in `recombination-rates', and let *bi* be
the sum *Σj<i ai\*di*.

The positions are then translated by linear functions:
*fi(x) = ai\*x + bi*,
such that for
positions *p*: *bpi<p<= bpi+1*
*p* is translated into *fi(p)*.

The input positions must be sorted.

## sort-markers

*Sort a list of markers.*

#### Prototype

```
(sort-markers marker-list)
```

#### Example

```
(sort-markers marker-list)
```

### Details

Sort a list of markers with relation to their position.

# (coasim rand)

*This module contains functions making random marker configurations.*

## make-random-ms-markers

*Make a list of random micro-satellite markers.*

#### Prototype

```
(make-random-ms-markers no-markers theta K)
```

#### Example

```
(define snp-markers (make-random-ms-markers 10 0.1 15))
```

### Details

Make a list of random positioned micro-satellite markers.
The theta and K parameters are similar to the `ms-marker'
function.

## make-random-positions

*Make a list of random positions.*

#### Prototype

```
(make-random-positions no-positions)
```

#### Example

```
(define positions (make-random-positions 10))
```

### Details

Makes a list of random positions between 0 and 1 (0 included, 1
excluded). The positions are returned sorted.

## make-random-snp-markers

*Make a list of random SNP markers.*

#### Prototype

```
(make-random-snp-markers no-markers low-freq high-freq)
```

#### Example

```
(define snp-markers (make-random-snp-markers 10 0.1 0.9))
```

### Details

Make a list of random positioned SNP markers. The low and
high frequency constraints are similar to the `snp-marker'
function.

## make-random-trait-markers

*Make a list of random trait markers.*

#### Prototype

```
(make-random-trait-markers no-markers low-freq high-freq)
```

#### Example

```
(define disease-marker (car (make-random-trait-markers 1 0.18 0.22)))
```

### Details

Make a list of random positioned trait markers. The low and
high frequency constraints are similar to the `trait-marker'
function.

# (coasim statistics)

*This module contains functions for calculating various statistics
on the ARG and on lists of haplotypes.*

## D

*Tajima's D statistics.*

#### Prototype

```
(D lst)
```

#### Example

```
(D '((0 1 1 1 1 0) (0 0 1 1 1 0) (0 1 1 0 1 0) (0 0 0 1 1 0)))
```

### Details

Calculates Tajima's D statistics on a list of haplotypes.

## S

*The number of mutations in a sample.*

#### Prototype

```
(S lst)
```

#### Example

```
(S '((0 1 1 1 1 0) (0 0 1 1 1 0) (0 1 1 0 1 0) (0 0 0 1 1 0)))
```

### Details

Calculates the number of mutations in a sample (calculated as the
number of informative sites).

## no-coalescence-events

*Returns the number of coalescence events in the ARG.*

#### Prototype

```
(no-coalescence-events arg)
```

#### Example

```
(define markers (make-random-snp-markers 10 0.1 0.9))
(define arg (simulate markers 100 :rho 400))
(define n (no-coalescence-events arg))
```

### Details

Returns the number of coalescence events in the ARG.

## no-gene-conversions

*Returns the number of gene conversions in the ARG.*

#### Prototype

```
(no-gene-conversions arg)
```

#### Example

```
(define markers (make-random-snp-markers 10 0.1 0.9))
(define arg (simulate markers 100 :gamma 10 :Q 0.2))
(define n (no-gene-conversions arg))
```

### Details

Returns the number of gene conversions in the ARG.

## no-recombinations

*Returns the number of recombinations in the ARG.*

#### Prototype

```
(no-recombinations arg)
```

#### Example

```
(define markers (make-random-snp-markers 10 0.1 0.9))
(define arg (simulate markers 100 :rho 400))
(define n (no-recombinations arg))
```

### Details

Returns the number of recombinations in the ARG.

## pi

*Calculates the average pairwise difference.*

#### Prototype

```
(pi list-of-haplotypes)
```

#### Example

```
(pi '((0 1 1 0) (1 0 1 0) (1 0 1 1)))
```

### Details

Calculates the average pairwise difference on a list of haplotypes.

## pi\_ij

*Calculates the number of differences between two haplotypes.*

#### Prototype

```
(pi_ij si sj)
```

#### Example

```
(pi_ij '(0 1 1 0) '(1 0 1 0))
```

### Details

Calculates the number of differences between two haplotypes.

## theta\_W

*Watterson's mutation rate estimate.*

#### Prototype

```
(theta_W lst)
```

#### Example

```
(theta_W '((0 1 1 1 1 0) (0 0 1 1 1 0) (0 1 1 0 1 0) (0 0 0 1 1 0)))
```

### Details

Calculates Watterson's mutation rate estimate.
